# Gamma-Secretase-Dependent and -Independent Effects of Presenilin1 on β-Catenin·Tcf-4 Transcriptional Activity

**DOI:** 10.1371/journal.pone.0004080

**Published:** 2008-12-30

**Authors:** Imma Raurell, Montserrat Codina, David Casagolda, Beatriz del Valle, Josep Baulida, Antonio García de Herreros, Mireia Duñach

**Affiliations:** 1 Unitat de Biofísica-CEB, Departament de Bioquímica i Biologia Molecular, Facultat Medicina, Universitat Autònoma de Barcelona, Bellaterra, Spain; 2 Programa de Recerca en Càncer, IMIM-Hospital del Mar, Barcelona, Spain; 3 Departament de Ciències Experimentals i de la Salut, Universitat Pompeu Fabra, Barcelona, Spain; University of Washington, United States of America

## Abstract

Presenilin1 (PS1) is a component of the γ-secretase complex mutated in cases of Familial Alzheimer's disease (FAD). PS1 is synthesized as a 50 kDa peptide subsequently processed to two 29 and 20 kDa subunits that remain associated. Processing of PS1 is inhibited by several mutations detected in FAD patients. PS1 acts as negative modulator of β-catenin·Tcf-4 transcriptional activity. In this article we show that in murine embryonic fibroblasts (MEFs) the mechanisms of action of the processed and non-processed forms of PS1 on β-catenin·Tcf-4 transcription are different. Whereas non-processed PS1 inhibits β-catenin·Tcf-4 activity through a mechanism independent of γ-secretase and associated with the interaction of this protein with plakoglobin and Tcf-4, the effect of processed PS1 is prevented by γ-secretase inhibitors, and requires its interaction with E- or N-cadherin and the generation of cytosolic terminal fragments of these two cadherins, which in turn destabilize the β-catenin transcriptional cofactor CBP. Accordingly, the two forms of PS1 interact differently with E-cadherin or β-catenin and plakoglobin: whereas processed PS1 binds E-cadherin with high affinity and β-catenin or plakoglobin weakly, the non-processed form behaves inversely. Moreover, contrarily to processed PS1, that decreases the levels of c-fos RNA, non-processed PS1 inhibits the expression c-myc, a known target of β-catenin·Tcf-4, and does not block the activity of other transcriptional factors requiring CBP. These results indicate that prevention of PS1 processing in FAD affects the mechanism of repression of the transcriptional activity dependent on β-catenin.

## Introduction

Presenilin 1 (PS1) encodes a ubiquitously expressed, eight-transmembrane protein involved in most cases of early-onset Familial Alzheimer's disease (FAD) [1], [2]. PS1 is synthesized as a 50 kDa polypeptide that is subject to endoproteolytic cleavage to generate stable N- and C-terminal derivatives of 29 and 20 kDa, respectively, which form the active 1:1 heterodimer [3]. As well as in ER and Golgi compartments, PS1 is located at the plasma membrane where it directly binds to the cadherin/catenin complexes [4]–[6].

PS1 assembles with nicastrin, aph-1 and pen-2 to form the large γ-secretase complex, responsible for the cleavage of several type-I transmembrane proteins, including the β-amyloid precursor protein (APP), Notch, CD44, ErbB4, E-cadherin and N-cadherin [5], [7]–[12], among others. The resulting intracellular proteolytic products (CTF2 in the case of cadherins) contain the cytosolic domains of the substrate proteins. Similar to the Notch intracellular domain, some of these peptides may have a role as regulators of gene expression [13]. Accordingly, work by Robakis and colleagues [10] has demonstrated that soluble N-cadherin-CTF2 binds the transcription factor CBP and promotes its degradation. Therefore, N-cadherin-CTF2 functions as a repressor of CBP-dependent transcription. Failed processing of PS1 and reduced cleavage of substrates has been detected in FAD patients carrying PS1 mutations; these PS1 mutants are deficient in the processing of Notch and N-cadherin [4], [14], [15]. It should be noted that PS1 is also involved in Wnt/β-catenin signaling, acting as a negative modulator of β-catenin·Tcf-4-mediated transcription [16]–[18]. β-catenin is a multifunctional protein initially described as a mediator of cadherin-dependent cell adhesion. In adherens junction complexes, β-catenin is required for recruiting the actin cytoskeleton, a role that can also be played by a related protein called plakoglobin or γ-catenin. Moreover, interaction of cadherins with p120-catenin that binds to a distinct site is necessary for the stabilization of E-cadherin at the cell membrane [19]. In addition to its function in cell adhesion, β-catenin is a central player in the Wnt pathway [20]–[22]. When released from the junction complex, β-catenin translocates to the nucleus, where it interacts with the Tcf-family of transcriptional factors and regulates the expression of a variety of genes involved in embryonic development and tumorigenesis [20], [21]. For instance, it has been shown that the activity of β-catenin·Tcf-4 is essential for maintaining the transcription of c-myc in intestinal cells and prevent cell arrest and premature cell differentiation [23]. The translocation of β-catenin to the nucleus is tightly controlled by the activity of a complex involved in β-catenin degradation. This complex includes the product of the tumor suppressor adenomatous polyposis gene, axin, and the Thr/Ser protein kinases, CKIα and glycogen synthase kinase 3β (GSK3β) [24]. As result of the activity of this complex, β-catenin is phosphorylated and degraded by the proteasome. The activity of the degradation complex is blocked by Wnt factors, which stabilize cytosolic β-catenin [20], [22]. Like β-catenin, plakoglobin also interacts with Tcf-4, but in a sub-domain other than that binding β-catenin. Since interaction of plakoglobin with Tcf-4 precludes binding of Tcf-4 to DNA, plakoglobin works as a negative regulator of the β-catenin-Tcf-4 complex [25]–[28].

In recent years, PS1 has been identified as an important negative regulator of β-catenin signaling. *Drosophila* presenilin (DPS) works as a negative modifier of wingless/Wnt in genetic screening [7] and mutants deficient in DPS accumulate armadillo/β-catenin in the cytoplasm [16]. Loss of PS1 in keratinocytes of knock-out mice causes high β-catenin-Tcf-mediated signaling, epidermal hyperplasia and tumors [18]. Moreover, PS1 deficiency in primary fibroblasts leads to increased activity of β-catenin target genes, including cyclin D1 [17]. However, the molecular basis of this inhibitory effect of PS1 on β-catenin-dependent transcription is controversial. According to some authors, PS1 down-regulates β-catenin activity by enhancing the stability of the E-cadherin-β-catenin complex [4], whereas other authors have not detected this effect [29]–[31]. In a previous study we reported that the inhibitory effect of non-processed presenilin (npPS1) on β-catenin signaling in epithelial cells does not depend on the proteolytic function of PS1 or the down-regulation of β-catenin [32]. In these cell lines PS1 binds to plakoglobin and enhances its interaction with Tcf-4, preventing Tcf-4 binding to DNA. Here, we have further extended these studies using MEFs deficient in the expression of PS1. Our data indicate that the processed and unprocessed PS1 forms decrease β-catenin·Tcf-4-dependent transcription through different mechanisms. Unlike npPS1, the inhibitory effect of processed presenilin (pPS1) on β-catenin activity is dependent on its γ-secretase activity, and requires binding of pPS1 to E- or N-cadherin and the subsequent cleavage of the cytosolic domains of these cadherins, which in turn destabilize the transcriptional cofactor CBP.

## Materials and Methods

### Preparation of DNA constructs

Full-length human PS1 and the point mutants M146L H163R, C410Y and deleted in exon 9 (Δe9), kindly provided by Dr. C.A. Saura (Institut de Neurociències, Universitat Autònoma de Barcelona, Spain) were cloned in pcDNA3.1 plasmid. Alternatively, full-length PS1 was inserted in the BamHI-EcoRV sites of pcDNA3.1*MycA*/His. The presence of the myc-tag at the C-terminal end of wild-type PS1 impedes the endoproteolytic processing of the protein. Full-length human CBP was cloned into the BamHI-NotI site of pcDNA3.1-HA. Full-length cDNA of murine E-cadherin was released from pBATEM2 plasmid by digestion with BglII-HindIII and cloned into BglII-HindIII-linearized pECFP-N1 vector. The DNA fragments corresponding to the cytosolic domain of E-cadherin (E-cadh-CTF2, amino acids 732–883) or N-cadherin (N-cadh-CTF2, amino acids 746–906) were amplified from murine RNA by RT-PCR using oligonucleotides corresponding to nucleotide sequences 2194–2219/2623–2649 and 2235–2253/2699–2717, respectively. The 0.5 kilobase amplification fragments were inserted in the BamHI-EcoRI site of a pGEX-6P3 plasmid for E-cadherin, or in the BamHI-XhoI site of a pGEX-6P1 plasmid for N-cadherin. To generate the eukaryotic expression plasmid, the cytosolic cDNA fragments of E- and N-cadherin were released from the pGEX-6P plasmid by digestion with BamHI-EcoRI for E-cadherin and with BamHI-XhoI for N-cadherin and cloned into BglII-EcoRI-linearized pEGFP-C1 or BamHI-XhoI-linearized pcDNA3.1, respectively. The preparation of the plasmids codifying for p120-catenin, Tcf-4 and β-catenin has been reported elsewhere [26], [33], [34].

### Expression of recombinant proteins and pull-down assays

GST-fusion proteins corresponding to the indicated forms of p120-catenin and the cytoplasmic domains of E-cadherin (E-cadh-CTF2) or N-cadherin (N-cadh-CTF2) were expressed in *E. coli* and purified by affinity chromatography on glutathione-Sepharose [35]. When indicated, GST-cadh-CTF2 were phosphorylated in a final volume of 50 µl of kinase buffer (9 mM MgCl_2_, 0.5 mM EGTA, 1 mM DTT, 0.5 mM EDTA, 28 mM α-glycerolphosphate pH 7.0, 0.1 mM ATP), using 22 pmols of GST-E-cadh-CTF2, GST-N-cadh-CTF2 or GST as a control, and 300 milliunits of protein kinase CKIα (Sigma). Reactions were performed for 40 min at 30°C. When required for the pull-down assays, GST was removed by cleaving with Pre-Scission protease (Amersham Biosciences). Pull-down assays were performed by using purified recombinant proteins fused to GST and cell extracts from MEF PS (+/+) cells or deficient for PS1 and PS2 (MEFs PS (−/−)), as described before [36]. Glutathione-Sepharose-bound proteins were analyzed by Western blotting with specific monoclonal antibodies (mAbs) against p120-catenin, β-catenin, E-cadherin, N-cadherin (all from BD Biosciences). The polyclonal antibody to the GST protein was from GE Healthcare. PS1 was analyzed with a polyclonal antibody that recognizes the full-length PS1 and the carboxyl-terminal PS1 fragment (Calbiochem). Serial immunoblots were performed after stripping the membranes. All binding assays were repeated three times. In order to quantify the amount of bound protein the autoradiograms were scanned in a densitometer.

### Transient cell transfections and analysis of protein expression

The cell lines SW-480 and murine embryonic fibroblast wild type (MEF PS (+/+)) or deficient for PS1 and PS2 (MEF PS (−/−)) were used in this study. MEF PS (−/−) were generously provided by Dr. B. De Strooper [37], [38]. SW-480 cells were established from a primary colon adenocarcinoma and express a mutant form of APC [39]. Cells were routinely grown in Dulbecco's modified Eagle's medium supplemented with 10% fetal calf serum. Transient expression of ectopic proteins was achieved in 80% of the confluent cells with the indicated cDNAs inserted in eukaryotic plasmid and using LipofectAMINE 2000 (Life Technologies) according to the instructions of the manufacturer. Cells were analyzed 24 or 48 hours after transfection. Total cell extracts were prepared from culture cells resuspended in lysis buffer (25 mM Tris-HCl, pH7.6, 150 mM NaCl, 1 mM EDTA, 1% digitonin), supplemented with protease inhibitors (0.3 mM aprotinin, 0.1 mM leupeptin, 1 µM pepstatin, 1 mM Pefabloc) and phosphatase inhibitors (10 mM NaF, 0.1 mM sodium orthovanadate). After passing cell homogenates 10 times by a insulin syringe, extracts were left on ice for 15 min and centrifuged at 14.000 xg for 5 min at 4°C. Supernatants constituted the whole cell extract.

### Cell fractionation and immunoprecipitation

Cytosolic fractions were prepared by homogenizing cells in RIPA buffer (25 mM Tris-HCl pH7.6, 200 mM NaCl, 1 mM EGTA, 0.5% sodium deoxycholate, 0.1% SDS, 1% Nonidet P-40), supplemented with protease inhibitors and phosphatase inhibitors. Cell homogenates were left on ice for 15 min and centrifuged at 500 xg for 10 min at 4°C to obtain the cytosolic fractions. Pellets were resuspended in the same volume of RIPA buffer supplemented with protease and phosphatase inhibitors. After passing cells 20 times by a insulin syringe, extracts were left on ice for 15 min and centrifuged at 20.000 xg for 10 min at 4°C. Supernatants constituted the nuclear fraction.

Proteins were immunoprecipitated from cell extracts (300 to 600 µg) by using 4 µg/ml of the appropriate antibody for 16 h at 4°C. Precipitated material was removed by centrifugation at 12,000 xg and the resulting supernatant was incubated for 90 min with 30 µl of protein A-agarose (Sigma). Immunoprecipitates were washed three times with lysis buffer and bound proteins directly eluted with electrophoresis sample buffer and analyzed by Western blotting. When appropriate, mouse TrueBlot reagent (BD Biosciences) was used as a secondary antibody in order to eliminate interferences in the autoradiogram.

### Luciferase reporter assays

Cells were transfected as above with the different promoters. TOP-Flash plasmid, a synthetic promoter sensitive to the activity of the β-catenin·Tcf-4 complex, that contains three copies of the Tcf-4 binding site upstream of a firefly luciferase reporter gene [40]. CRE-dependent transactivation was measured in the presence of a CRE-luciferase reporter plasmid (pCRE-Luc) (Oncogene), and NFκB-dependent transactivation was measured with the NF3 plasmid, a NFκB-sensitive plasmid containing three binding sequences for this transcriptional factor upstream a luciferase reporter gene, provided by Dr. M. Fresno (Universidad Autónoma de Madrid, Spain). PS1, CBP, E-cadh-CTF2, N-cadh-CTF2, Tcf-4, p120-catenin or the indicated plasmids and controls were cotransfected when indicated. Activity of the product of the *Renilla* luciferase gene under the control of a constitutive thymidine kinase promoter (Promega) was used as a control. Assays were always performed in triplicate; the average of the results of three-four independent transfections±S.D. is given.

### Statistical analysis

Statistical analyses were carried out using SPSS software version 14. The results of the means percentage were compared by ANOVA. *P* values of <0.05 were considered to be significant in each case.

### Stable cell transfections and cell proliferation analysis

MEF PS (−/−) cells were cotransfected with 10.5 µg of either pcDNA3.1-PS1 (pPS1), pcDNA3.1-*Myc/His-*PS1 (npPS1) or empty vector and 1.5 µg pBABE-puro hrGFP. After 48 h, transfected cells were selected with medium containing 2.5 µg/ml Puromycin for additional 48 hours. Total cell protein extracts were prepared as indicated above; RNAs were obtained as previously reported [41] and analysed by quantitative RT-PCR using the QuantiTect SYBR Green RT-PCR (Qiagen) in triplicate using oligonucleotides specific for c-Fos (5’-TCCAGCATGGGCTCGC-3’, sense and 5’-GACCGTGGGAATGAAGTTGG-3’, antisense), c-Myc, Fibronectin or HPRT [41]. Cell proliferation was determined counting every 24 h up to 96 h post-seeding the number of cells in 20 different colonies of each subpopulation. The proliferation rate of cells transfected with pPS1 and npPS1 was expressed referred to the proliferation rate of control cells in terms of percentage.

## Results

**Figure 1:**
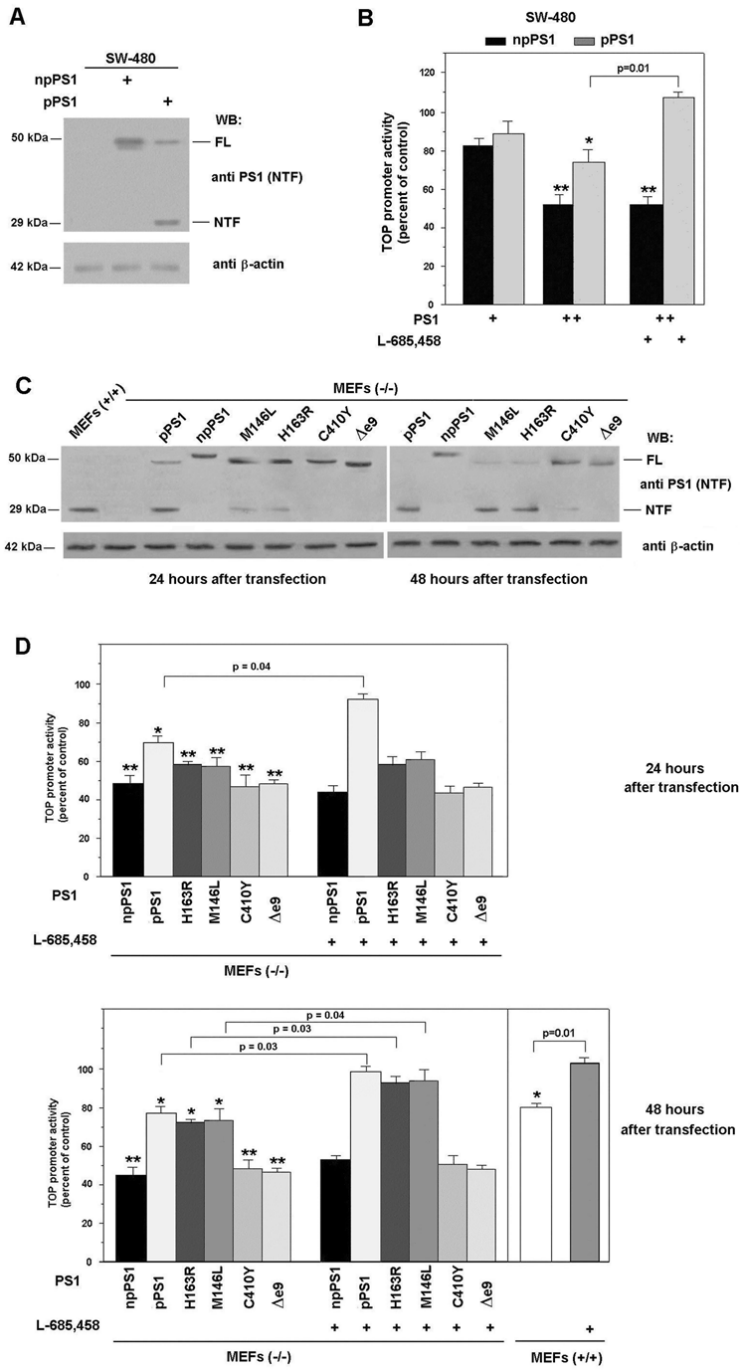
Processed and non-processed PS1 block β-catenin·Tcf-4-dependent transcription differently. SW-480 (A,) and MEF PS (−/−) cells (C) were transfected with 5 µg of pcDNA3 plasmid containing either *Myc/His*-tagged PS1 (npPS1), wild-type PS1 (pPS1), the indicated mutants, or empty vector as a control. After 48 hours (A) or at the indicated times (C), cell extracts were prepared as described in *Materials and Methods*. 50 µg of untransfected or transfected total cell extracts were analyzed by SDS-PAGE and Western blot with antibodies anti-PS1 (amino acids 1–65) and anti-β-actin as a control (A and C). In panels B and D cells SW-480 and MEFs were cotransfected with 150 (+, in B) or 300 ng (++, in B; and D) of pcDNA-3 containing npPS1, pPS1, or, the indicated mutants, plus TOP-FLASH (50 ng) and pTK-*Renilla* (10 ng) luciferase plasmids. Relative luciferase activity was determined with a dual luciferase reporter assay system 48 hours after transfection (B) or at the indicated times (D), and the result was normalized using the *Renilla* luciferase activity for each sample. Percentage activity was calculated by comparing levels of luciferase activity with levels after transfection of the empty plasmid alone. 5 µM γ-secretase inhibitor L-685,458 (Calbiochem) was added to the medium for the last 24 h (panels B and D). Values are the average +/− S.D. of three-four experiments performed in triplicate. One asterisk (*) indicates p<0.05; two asterisks (**) p<0.01; in the rest of the comparisons, the value of p is presented.

We analyzed the effect of two different forms of PS1 on the activity of TOP reporter plasmid, a widely used system to determine β-catenin·Tcf-4 transcriptional activity. An untagged form of PS1 was partially processed when transfected to epithelial intestinal SW-480 cells (Figure 1A). However, a form of PS1 containing a myc tag in the C-terminus was not proteolysed (Figure 1A) and remained as a 50 kDa protein, as previously shown [42]. Both PS1 forms inhibited TOP activity, although npPS1 did so to a greater extent than processed pPS1 (Figure 1B). More importantly, the inhibition of TOP activity by both forms of PS1 was differently regulated by the γ-secretase inhibitor L-685,458 [43]: whereas this compound did not affect the repression by npPS1, it totally prevented the effect of pPS1 (Figure 1B).

Similar experiments were performed in murine embryo fibroblasts (MEFs) obtained from PS1 and PS2 knock-out animals, transfecting the two PS1 forms to levels similar to those detected in control MEFs (+/+) (Figure 1C). The tagged PS1 (npPS1) was not processed in these PS1 (−/−) cells at the two time points analyzed. Conversely, wild-type PS1 was detected mainly as a 29 kDa band 24 hours after transfection and was totally processed after 48 hours. We also determined the behavior of four PS1 mutants, initially described in FAD patients. Two of these mutants, M146L and H163R, were processed although not as efficiently as the wild-type form, since the predominant form at 24 hours was the 50 kDa uncleaved protein (Figure 1C). However at 48 hours they were almost completely processed. On the other hand, C410Y and Δe9 PS1 mutants remained as a 50 kDa band at the two time points analyzed (Figure 1C).

The effect of all these PS1 forms on the TOP activity was also determined. Although both the processed and non-processed forms of PS1 inhibited this activity, the non-processed proteins (npPS1, C410Y and Δe9) did it to a higher extent (Figure 1D). This difference was more easily observed after 48 hours (Figure 1D, lower panel), correlating with the more extensive processing of the wild type, M146L and H163R PS1 forms. Moreover, the inhibition of the TOP activity by the non-processed forms (thus, npPS1, C410Y and Δe9 at 48 hours; all but the wild-type protein at 24 hours) was insensitive to the γ-secretase inhibitor L-685,458, whereas that of the processed forms (wild-type pPS1 or M146L and H163R mutants) was prevented by this compound (Figure 1D). The same sensitivity of TOP activity to L-685,458 was observed in wild-type MEFs (+/+); addition of this compound also raised the activity of this promoter in wild-type MEFs up to the values detected in MEFs (−/−) (Figure 1D, lower panel).

Therefore, these results indicate that PS1 presents γ-secretase-dependent and -independent inhibitory effects on β-catenin·Tcf-4-dependent transcription that are related to its own processing.

**Figure 2:**
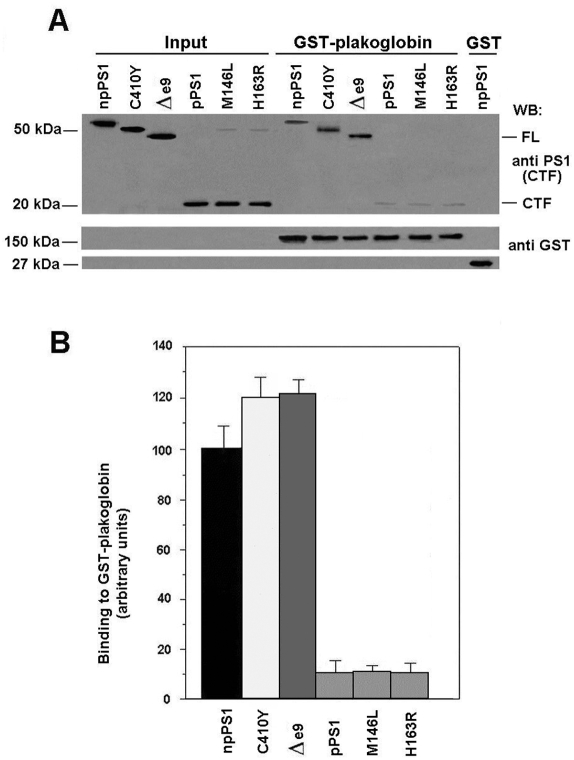
npPS1 shows a higher binding to plakoglobin than pPS1. (A) Pull-down assays were performed by incubating 10 pmols of GST-plakoglobin or GST (as a control) with 500 µg of whole-cell extracts from MEFs PS (−/−) transfected with the indicated froms of PS1 inserted in pcDNA3 plasmid. Protein complexes were affinity-purified with glutathione-Sepharose and analyzed by SDS-PAGE and Western blotting with anti-PS1 (aa 1–65) and anti-GST, to ensure that similar levels of fusion proteins were present in all cases. In the Input lane, a sample corresponding to 5% of the total cell extracts used for the assay was loaded. (B) Western blots corresponding to three different experiments were scanned and quantified with respect to the corresponding input. The panel shows the average (+/− S.D.) of the binding calculated for the different PS1 forms.

Inhibition of β-catenin·Tcf-4 transcriptional activity by npPS1 is related to its ability to interact with plakoglobin [32]. Using pull-down assays with GST-plakoglobin as bait, pPS1 showed lower binding to this protein than npPS1 (Figure 2A). A quantification of the association of both PS1 forms with GST-plakoglobin indicated that npPS1 binds ten-fold better than pPS1 (Figure 2B). No significant differences were observed in the association with GST-plakoglobin for the different non-processed mutants used in Figure 1, and the wild-type non-processed protein (Figure 2B). The two processed PS1 mutants (M146L and H163R) presented a similar binding to GST-plakoglobin than wild-type pPS1. Analogous results were obtained when binding to β-catenin was analyzed (not shown). These results suggest that pPS1 does not affect β-catenin·Tcf-4 transcriptional activity through its binding to plakoglobin or β-catenin and corroborate that the two PS1 forms act through different mechanisms.

**Figure 3:**
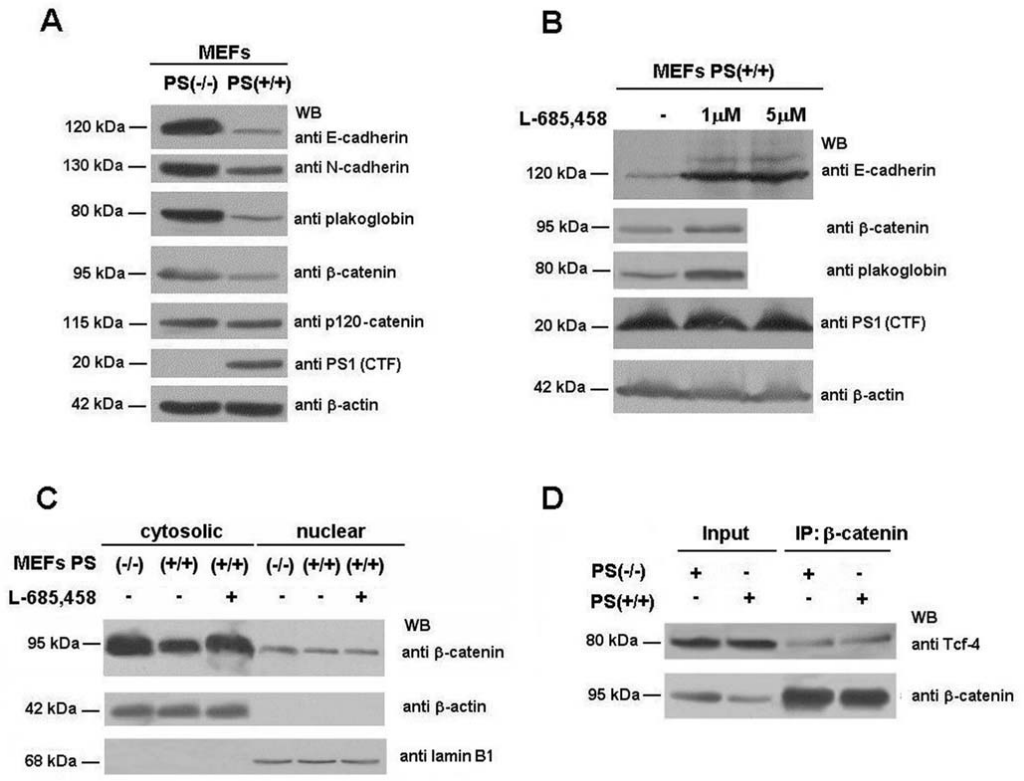
PS depletion increases protein levels of adherens junction-associated proteins. (A) 50 µg of MEF PS (−/−) and PS (+/+) total-cell extracts were analyzed by Western blotting with the indicated antibodies. (B) MEF PS (+/+) cells were incubated with 1–5 µM γ-secretase inhibitor L-685,458 for 24 h. Then, total cell extracts were prepared as described in *Experimental Procedures* and analyzed by SDS-PAGE and Western blot with the indicate antibodies. No effect of the γ-secretase inhibitor was detected in PS/−/−) cells on the levels of the studied proteins (not shown). (C) Cytosolic and nuclear fractions of MEF PS (−/−) and PS (+/+) cells were prepared as described. When indicated, MEF PS (+/+) cells were incubated with 5 µM γ-secretase inhibitor L-685,458 for 24 h. 50 µg of each fraction was analyzed by Western blotting with anti-β-catenin, anti-β-actin and anti-laminB1. (D) 800 µg of MEF PS (−/−) and PS (+/+) total-cell extracts were immunoprecipitated with an anti-β-catenin MAb, and analyzed by Western blot with specific antibodies against Tcf-4 and β-catenin. In the Input lane, a sample corresponding to 7% of the total cell extracts used for the assay was loaded.

We further investigated the mechanism involved in the repression of β-catenin·Tcf-4 transcriptional activity by pPS1 in MEFs. Deficiency of PS1 and PS2 caused increased levels of E-cadherin, N-cadherin, plakoglobin and, to a lesser extent, β-catenin (Figure 3A). The expression of another adherens junction-associated protein, p120-catenin, was not altered. The differences in the expression of these proteins were not due to different transcription since their RNAs remained equal (not shown). Addition of the γ-secretase inhibitor L-685,458 reverted the levels of these proteins (Figure 3B), suggesting that altered expression of these proteins might be the origin of the repression of β-catenin·Tcf-4 transcriptional activity.

It has been proposed that PS1 promotes the degradation of β-catenin and, as a consequence of lower availability of this protein, inhibits β-catenin translocation to the nucleus and its transcriptional activity [44]. However, the decrease in β-catenin levels, when comparing wild-type and PS-deficient MEFs, was detected in the cytosol but not in the nucleus (Figure 3C). Moreover, in both cell types the amount of β-catenin immunoprecipitated with Tcf-4 was similar (Figure 3D), indicating that a mechanism other than decreased expression of β-catenin is responsible for the effect of processed PS1 on β-catenin-transcriptional activity in MEFs.

**Figure 4:**
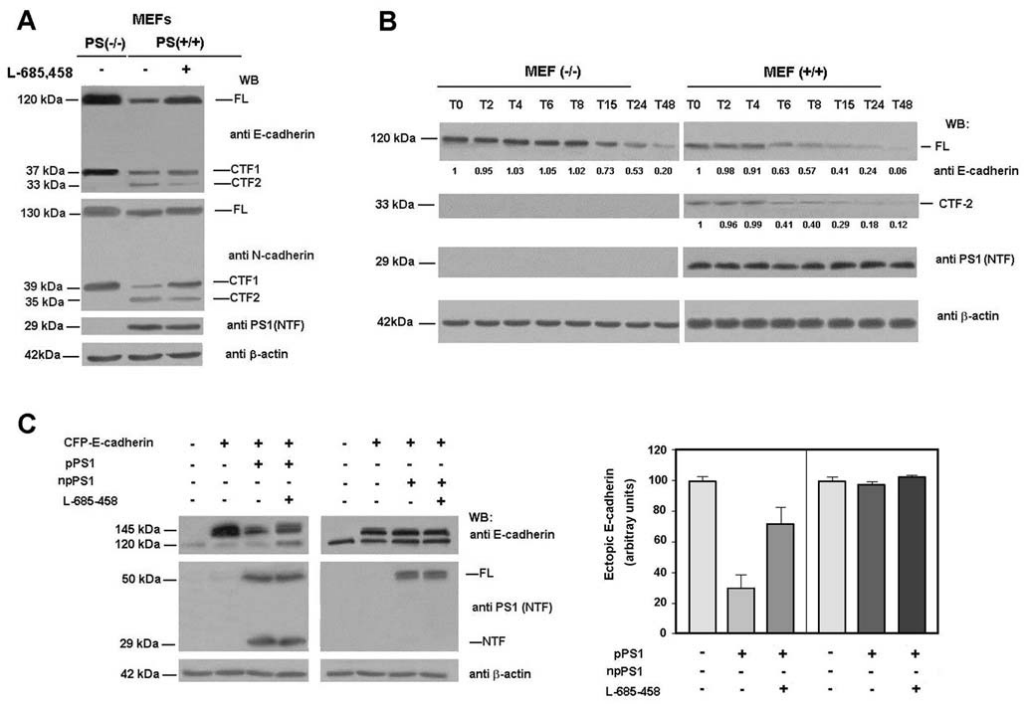
pPS1, and not npPS1, is responsible for down-regulation of E-cadherin levels. (A) MEF PS (+/+) cells were incubated with 5 µM γ-secretase inhibitor L-685,458 for 24 h. 50 µg of MEFs PS (−/−) and PS (+/+) total cell extracts were analyzed by SDS-PAGE and Western blot with the indicated antibodies. (B) MEF PS (−/−) and PS (+/+) cells were incubated with 25 µg/ml cycloheximide for the indicated time periods. 10 µg of MEF PS (−/−) and 60 µg of MEF PS (+/+) total cell extracts were analyzed by Western blot with anti-E-cadherin, anti-PS1 (N-terminus) and anti-β-actin-specific antibodies. Autoradiograms were scanned; the values obtained for E-cadherin were referred to the initial time. (C) MEF PS (−/−) cells were cotransfected with 2.5 µg of pECFPN1-E-cadherin and 2.5 µg of pcDNA3.1-*Myc/His*-PS1 (npPS1) or pcDNA3.1-PS1 (pPS1) plasmids. 24 h after transfection, 5 µM γ-secretase inhibitor L-685,458 was added to the medium for 24 h. Percentage of transfection was estimated to be approximately 80%, determining the number of CFP-positive cells. 50 µg of total cell extracts were analyzed by Western blotting with the indicated antibodies. At the right, the ectopic E-cadherin levels of three different experiments were quantified and represented with respect to control (no expression of either pPS1 or npPS1). Values are the average +/− S.D. of these experiments.

PS1-deficient MEFs showed higher levels of E- and N-cadherin than wild-type MEFs. For both cadherins, full-length proteins and C-terminal fragments, CTF1 (at 37 kDa for E-cadherin and 39 kDa for N-cadherin) were detected at higher levels in MEFs (−/−) than in MEFs (+/+) (Figure 4A, lanes 1 and 2). However, MEFs (−/−) lack the 35 kDa C-terminal fragments (CTF2) generated by the action of γ-secretase on CTF1 (Figure 4A). Addition of L-685,458 increased the amount of full-length and CTF1 E-cadherin in wild-type MEFs and down-regulated the CTF2 fragment (Figure 4A, compare lanes 2 and 3). A more detailed study of the stability of E-cadherin in MEFs demonstrated that the absence of PS increased the half-life of this protein from 8 hours in wild-type MEFs to 24 hours in PS-deficient cells (Figure 4B).

The destabilization of E-cadherin by PS1 was also observed when both proteins were ectopically expressed in MEFs (−/−). As shown in Figure 4C, the amount of ectopic CFP-E-cadherin, detected as a 145 kDa band, was down-regulated by cotransfection with pPS1. Levels of E-cadherin were partially recovered by addition of the γ-secretase inhibitor. However, transfection of npPS1 did not modify the stability of E-cadherin (Figure 4C).

**Figure 5:**
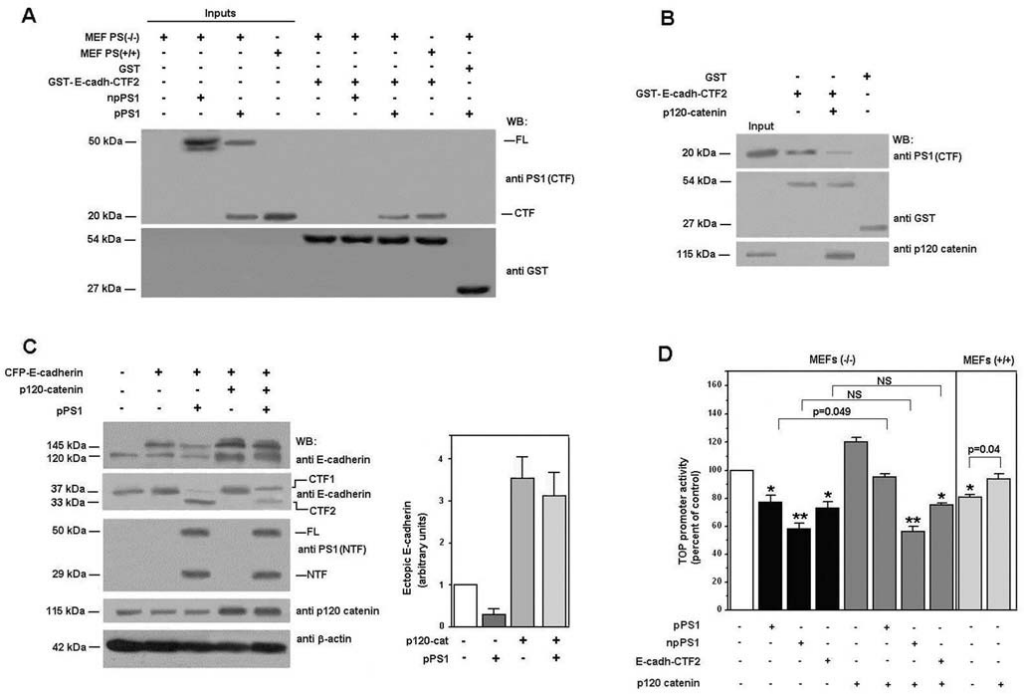
p120-catenin prevents the interaction of pPS1 with E-cadherin, the generation of E-cadherin-CTF2 fragment and the inhibition of β-catenin·Tcf-4 transcriptional activity. (A) MEF PS (−/−) cells were transfected with npPS1 or pPS1: pcDNA3.1-*Myc/His*-PS1 (npPS1) or pcDNA3.1-PS1 (pPS1). After 48 h total cell extracts were prepared and incubated with 15 pmols of GST-E-cadh/CTF2 or GST as a control; cell extracts from MEFs PS (+/+) were also incubated. Protein complexes were purified with glutathione-Sepharose and associated PS1 was analyzed by Western blotting with a specific antibody against PS1 (C-terminus). Blots were re-analyzed with anti-GST to ensure that similar levels of fusion protein forms were present in all samples. In the Input lane, a sample containing 5% of the total cell extracts used for the assay was loaded. (B) 12 pmol of GST-E-cadh-CTF2 or GST as a control were pre-incubated with 20 pmols of p120-catenin and pulldown assays performed as above with 1 mg of MEF PS (+/+) total cell extracts. (C) MEF PS (−/−) cells were cotransfected with 2.5 µg of pECFPN1-E-cadherin and pcDNA3.1-PS1 (pPS1) or pcDNA3.1-p120-catenin. 50 µg of total cell extracts were analyzed by SDS-PAGE and Western blot with the indicated antibodies. At the right, the ectopic E-cadherin levels of three different experiments were quantified and represented with respect to control (no additions). Values are the average +/− S.D of these experiments. (D) MEF PS1(−/−) cells were cotransfected with pcDNA3.1-PS1 (pPS1), pcDNA3.1-*Myc/His*-PS1 (npPS1), pcEGFPC1-E-cadh-CTF2, pcDNA3.1-p120-catenin or empty vector (150 ng), TOP-FLASH (50 ng) and pTK-*Renilla* (10 ng) luciferase plasmids. MEF PS1(+/+) cells were transfected with pcDNA3.1-p120-catenin or empty vector (150 ng). Relative luciferase activity was determined with a dual luciferase reporter assay system 48 h after transfection, and the result was normalized using the *Renilla* luciferase activity for each sample. Percentage activity was calculated by comparing levels of luciferase activity with levels after transfection of the empty plasmid alone. Values are the average +/− S.D. of three-four experiments performed in triplicate. −, absent; +, present. (*) indicates p<0.05; (**), p<0.01; in the rest of the comparisons, the p value is shown. NS, not significant.

The direct interaction of E-cadherin with the processed and non-processed PS1 forms was also determined by pull-down assays. As observed in Figure 5A (lanes 7 and 8), binding of pPS1 to the cytosolic fragment of E-cadherin was detected, either when pPS1 was transfected or when the endogenous protein was present in MEFs. However, npPS1 did not interact with E-cadherin, although this form was expressed at higher levels than pPS1 (Figure 5A).

It has been reported that PS1 associates with E-cadherin at the p120-catenin binding site [4]. Accordingly, interaction of pPS1 with E-cadherin competed with p120-catenin, since addition of this recombinant protein decreased the amount of PS1 associated with GST-E-cadh-CTF2 (Figure 5B). Moreover, transfection of p120-catenin to MEF (−/−) cells stabilized the ectopically expressed E-cadherin (CFP-E-cadherin, corresponding to the 145 kDa band) or the endogenous protein (corresponding to the 120 kDa band) and totally prevented the decrease in the levels of these proteins caused by simultaneous expression of pPS1 (Figure 5C, compare lanes 3 and 5). As expected, p120-catenin also inhibited the generation of E-cadherin CTF2 observed after expression of pPS1 (Figure 5C).

Next, we checked the influence of p120-catenin on the effect of PS1 on β-catenin·Tcf-4 transcriptional activity. Addition of p120-catenin prevented the repression caused by pPS1 of TOP promoter activity without affecting that caused by npPS1 (Figure 5D), suggesting again that the generation of E-cadherin or N-cadherin CTF2 is involved in the effect of pPS1, but not of npPS1, on β-catenin·Tcf-4-dependent transcription. Accordingly, transfection of E-cadh-CTF2 (Figure 5D) and N-cadh-CTF2 (not shown) inhibited TOP promoter activity. As expected, repression by E-cadh-CTF2 was independent of p120-catenin (Figure 5D, bars 4 and 8).

Subcellular location of CTF2, determined transfecting this protein tagged with GFP, indicated that this protein was detected exclusively in the cytosol (data not shown). This result, and previous observations shown in Figures 3C and 3D (pPS1 does not prevent β-catenin translocation to the nucleus or binding to Tcf-4), suggest that the effect of CTF2 is other than inhibition of β-catenin binding to Tcf-4.

**Figure 6:**
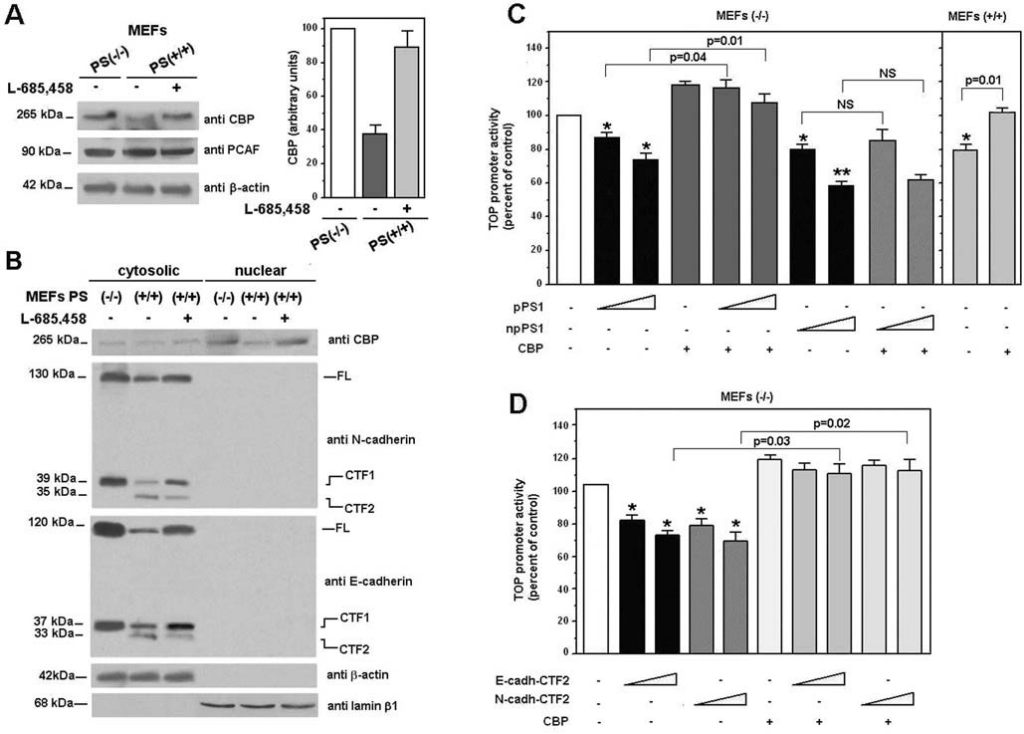
CBP prevents the repression of β-catenin·Tcf-4 transcriptional activity caused by the expression of pPS1, E- or N-cadherin CTF2. (A) MEF PS (+/+) cells were incubated with 5 µM γ-secretase inhibitor L-685,458 for 24 h. 50 µg of MEFs PS (−/−) and PS (+/+) total cell extracts were analyzed by SDS-PAGE and Western blot with anti-CBP and anti-β-actin antibodies. Autoradiograms were scanned and the average level of CBP obtained in the three different experimental conditions is represented in the right panel. Values correspond to the average +/− S.D of four experiments. (B) Cytosolic and nuclear-associated fractions of MEF PS (−/−) and PS (+/+) cells (incubated with 5 µM γ-secretase inhibitor L-685,458) were prepared as detailed in *Experimental Procedures*. 50 µg of each fraction was analyzed by SDS-PAGE and Western blot with the indicated antibodies. (C) MEF PS (−/−) cells were cotransfected with either pcDNA3.1-PS1 (pPS1) or pcDNA3.1-*Myc/His*-PS1 (npPS1) and pcDNA3.1-HA-CBP and (D) with pcEGFPC1-E-cadh-CTF2 or pcDNA3.1-N-cadh-CTF2 or empty vector (150–300 ng), TOP-FLASH (50 ng) and pTK-*Renilla* (10 ng) luciferase plasmids. Relative luciferase activity was determined with a dual luciferase reporter assay system 48 h after transfection, and the result was normalized using the *Renilla* luciferase activity for each sample. Percentage activity was calculated by comparing levels of luciferase activity with levels after transfection of the empty plasmid alone. Values are the average +/− S.D. of three-four experiments performed in triplicate. (*) indicates p<0.05; (**), p<0.01; in the rest of the comparisons, the p value is shown. NS, not significant.

N-cadherin CTF2 is involved in the down-regulation of CBP [10], a transcriptional coactivator required for β-catenin transcriptional activity. CBP expression was higher in PS-deficient MEFs than in the normal counterpart cells (Figure 6A). However, the levels of another transcriptional factor of this family, pCAF, were not altered. Addition of L-685,458 raised CBP in wild-type MEFs up to levels similar to those detected in MEFs (−/−) (Figures 6A and 6B). Although mostly nuclear, CBP was also detected in the cytosol in lower amounts (Figure 6B), where E-cadh-CTF2 was also present.

Supplementation with exogenous CBP prevented the repression of β-catenin·Tcf-4-dependent transcription caused by expression of pPS1 in MEFs (−/−) (Figure 6C) and stimulated this parameter in MEFs (+/+). On the other hand, expression of CBP did not prevent the inhibition of TOP activity caused by npPS1 (Figure 6C). A similar effect was observed when the repression of TOP promoter by E- or N-cadherin CTF2 was analyzed: the effect of both cadherin fragments was prevented by CBP over-expression (Figure 6D).

**Figure 7:**
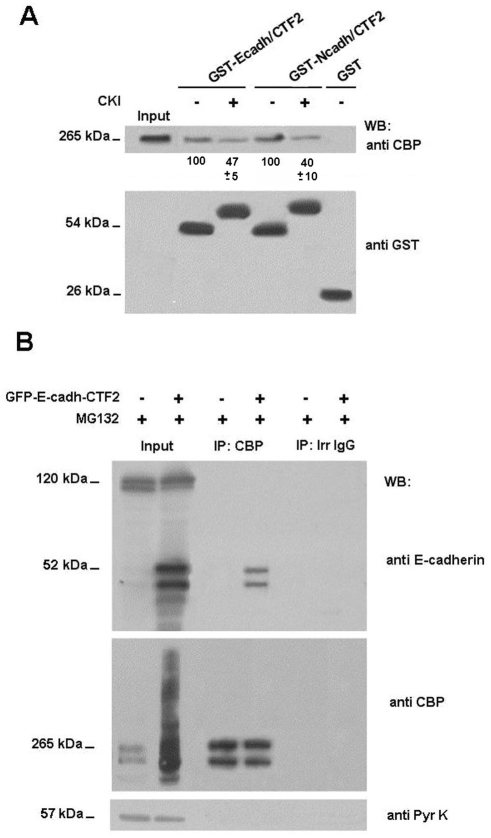
The CTF2 fragment of E-cadherin interacts with CBP. (A) GST-E-cadh-CTF2 and GST-N-cadh-CTF2 were phosphorylated with CKIδ, as described in *Material and Methods*. 20 pmols of non-phosphorylated or phosphorylated GST-cadh-CTF2 proteins, or GST as a control, were incubated with 1.2 mg of MEF PS (−/−) total cell extracts. Protein complexes were affinity-purified with glutathione-Sepharose and analyzed by SDS-PAGE and Western blotting with anti-CBP and anti-GST, to ensure that similar levels of fusion proteins were present in all cases. In the Input lane, a sample corresponding to 5% of the total cell extracts used for the assay was loaded. Autoradiograms were scanned and the average of CBP bound with respect to the value obtained without CKI is shown below the western blot (average +/− S.D. of three experiments). (B) MEFs PS (−/−) were transfected with 7 µg of GFP or E-cadh-CTF2-GFP, to simulate the CTF2 fragment present in MEF PS1/PS2 (+/+). After 24 h expression, cells were treated with the proteosome inhibitor MG132 at 30 µM for 4 h. RIPA buffer was used to obtain cytosolic cell fraction. For the IP, 500 µg of the cytosolic fraction were incubated with 2.5 µg of anti CBP antibody to a final lysate concentration of 1 µg/µl. 30 µg of cytosolic cell lysates were loaded for inputs.

Down-regulation of CBP levels by N-cadherin-CTF2 has been reported to be dependent on their interaction [10]. Association of CBP was detected not only with N-cadherin CTF2 but with E-cadherin CTF2. Fusion proteins containing E-cadherin or N-cadherin CTF2 showed similar binding to CBP (Figure 7A). Phosphorylation of CTF2 by CK1, a protein kinase active on this fragment [45], consistently decreased the association of CBP with CTF2, raising the possibility that this interaction might be modulated *in vivo.* The interaction was also detected by co-immunoprecipitation using cytosolic fractions and a previous treatment with proteasome inhibitors such as MG132, in order to prevent the degradation of CBP. As shown in Figure 7B (lane 2), in extracts treated with MG132, transfection of E-cadherin CTF2 induced the appearance of high molecular weight forms of CBP, presumably corresponding to ubiquitinated variants of this factor. The presence of CTF-2 was detected in CBP immunoprecipitates, further demonstrating the association between these two proteins (Figure 7B).

**Figure 8:**
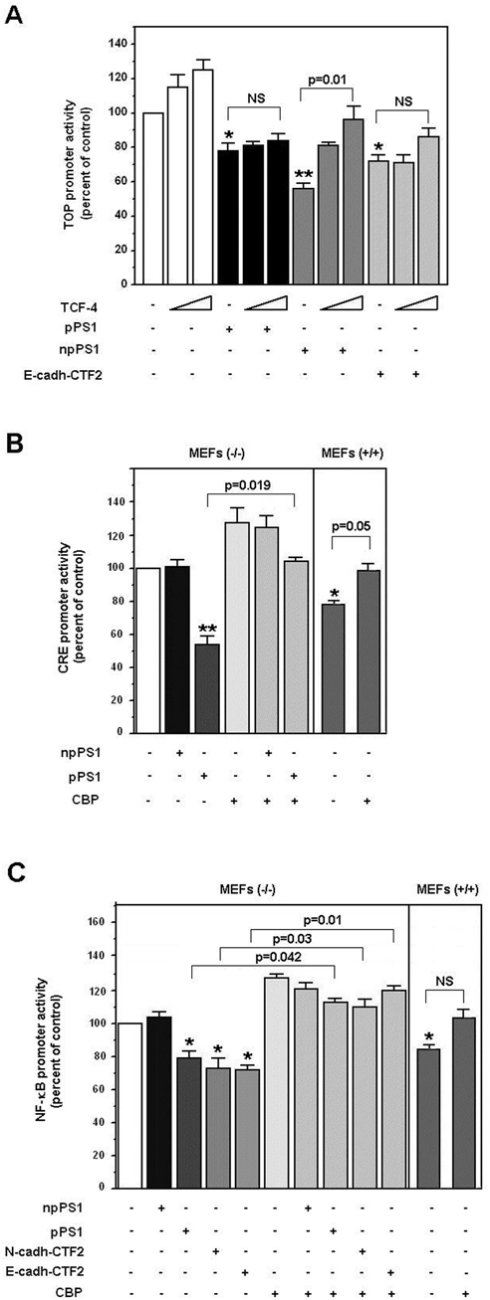
Transcriptional repression by npPS1 and pPS1 has different selectivity and requirements. (A) MEF PS (−/−) cells were cotransfected with pcDNA3.1-Tcf-4 (150 and 300 ng), pcDNA3.1-PS1 (pPS1), pcDNA3.1-*Myc/His*-PS1 (npPS1), pcEGFPC1-E-cadh-CTF2 or empty vector (150 ng), TOP-FLASH (50 ng) and pTK-*Renilla* (10 ng) luciferase plasmids. (B and C) MEF cells were cotransfected with 250 ng of pcDNA3.1-PS1 (pPS1), pcDNA3.1-*Myc/His*-PS1 (npPS1), pcEGFPC1-E-cadh-CTF2, pcDNA3.1-N-cadh-CTF2, pcDNA3.1HA-CBP or empty vector, and pCRE-luc (60 ng) (panel B) or NF-κB-dependent promoter (50 ng) (panel C) and pTK-*Renilla* (10 ng) luciferase plasmids. Relative luciferase activity was determined with a dual luciferase reporter assay system 48 h after transfection, and the result was normalized using the *Renilla* luciferase activity for each sample. Percentage activity was calculated by comparing levels of luciferase activity with levels after transfection of the empty plasmid alone. Values are the average +/− S.D. of three-four experiments performed in triplicate. (*) indicates p<0.05; (**), p<0.01; in the rest of the comparisons, the p value is shown. NS, not significant (p>0.05).

**Figure 9:**
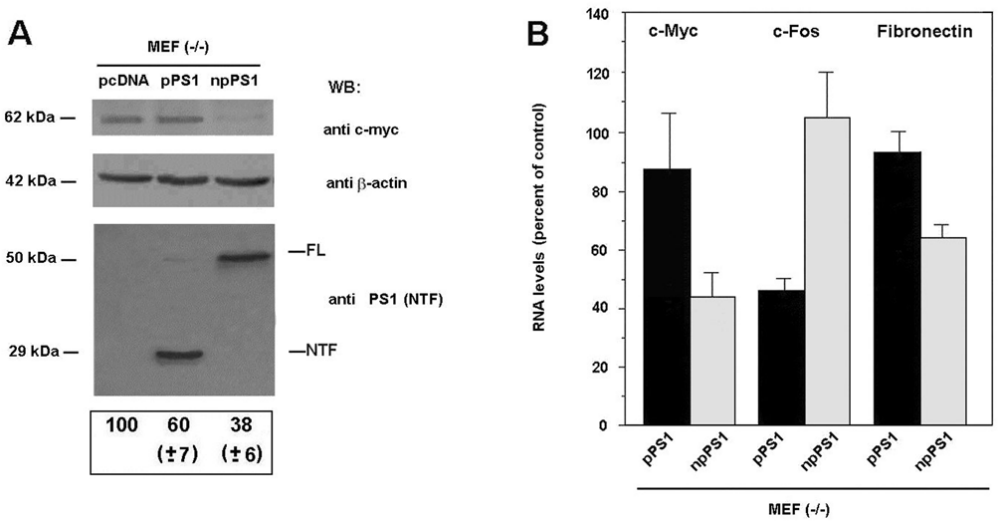
pPS1 and npPS distinctly affect the expression of β-catenin·Tcf-4-dependent transcriptional targets. MEF PS (−/−) cells were cotransfected with 10.5 µg of either pcDNA3.1-PS1 (pPS1), pcDNA3.1-*Myc/His-*PS1 (npPS1) or empty vector and 1.5 µg pBABE-puro hrGFP. After 48 h, transfected cells were selected with 2.5 µg/ml Puromycin for 48 h and protein extracts or RNAs were prepared as described in *Material and Methods.* (A) 25 µg of total cell extracts were analyzed by SDS-PAGE and Western blot with antibodies against PS1 (1–65), c-myc and β-actin as a control. 10^5^ transfected cells were seeded and cell proliferation analyzed by counting the number of cells every 24 h. The reduction in the proliferation rate of pPS1 and npPS1 transfected cells is shown referred to the proliferation rate of cells transfected with the empty vector. (B) RNAs were obtained from the three transfectants populations as reported in Methods and the levels of the indicated transcripts analyzed by quantitative RT-PCR. The panel shows the relative expression of these genes with respect to the control (average +/− S.D. of three determinations carried out in triplicate).

We also analyzed several different properties of the transcriptional repression by processed and non-processed PS1. Since npPS1 affects β-catenin·Tcf-4 transcriptional activity by sequestering Tcf-4 in non-functional complexes, inhibition by this PS1 form is prevented by Tcf-4 over-expression [32]. These results were reproduced in MEFs (−/−) (Figure 8A, lanes 7–9). However, neither pPS1 nor E-cadherin CTF2 inhibition of TOP activity were significantly affected by Tcf-4 (Figure 9A, lanes 4–6 for pPS1, and 10–12 for CTF2). According to these results, the repression of TOP activity by the non-processed PS1 mutants (C410Y or Δe9), but not by the processed forms (H163R and M146L), was also prevented by over-expression of Tcf-4 (data not shown).

Since pPS1 decreases the cell levels of CBP, we expected that it not only affects the activity of β-catenin·Tcf-4 complex but also that of other transcriptional factors dependent on this cofactor, such as CREB (cyclic AMP response-element binding protein). Binding of CBP is required for stimulation by CREB of the expression of genes containing CREs (cyclic AMP responsive elements) in their promoters. Therefore, we checked the effects of both PS1 forms on the activity of a CRE luciferase reporter plasmid in cells stimulated with forskolin. Whereas npPS1 did not affect the activity of this promoter, pPS1 repressed it by 50% (Figure 8B). As expected, expression of CBP reversed the inhibition of CRE activity by pPS1. Activity of this promoter was also significantly lower in wild-type MEFs than in PS-deficient cells and was recovered by CBP expression (Figure 8B).

CBP also modulates the activity of NF-κB [46]. Activity of an NF-κB-reporter plasmid was also sensitive to the expression of pPS1, E-cadherin or N-cadherin CTF2, but not to npPS1 (Figure 8C). Mimicking the results obtained with TOP or CRE reporters, co-expression of CBP abolished the inhibition by these three factors. Activity of this promoter was lower in wild-type MEFs than in mutant cells and was increased, although not statistically significantly, by CBP expression.

Finally, we analyzed the effect of the two PS1 forms on gene expression in stable MEFs transfectants. Both forms differently affected cell proliferation: whereas MEF(−/−) cells expressing npPS1 proliferated at a rate 38% of control cells, those expressing pPS1 did it at a 60% (Figure 9A). Since c-myc is a well-established target of β-catenin·Tcf-4 transcriptional activity [23], [47], we analyzed whether processed and non-processed PS1 forms similarly inhibited the expression of this protein. As shown in Figure 9A, npPS1 greatly decreased the expression of c-myc, whereas pPS1 barely affected it.

The effect of both PS1 forms on c-Fos RNA was also examined, since expression of this gene has been reported to be sensitive to CREB, pPS1 and the γ-secretase inhibitor L-685,458 [10]. As shown in Figure 9B, pPS1 transfectants contain lower levels of c-Fos RNA than control populations; however expression of npPS1 did not substantially modify this RNA. The effect on c-myc RNA was the opposite, in agreement with the results of c-myc protein analysis: npPS1 substantially decreased c-myc RNA whereas pPS1 did not (Figure 9B). Similar effects were obtained on Fibronectin RNA, another target of β-catenin [48], although in this case the repression caused by npPS1 was smaller (Figure 9B).

Therefore these results indicate that processed and non-processed PS1 act differently on gene transcription.

## Discussion

In recent years, a growing amount of evidence has indicated that PS1 controls the transcriptional activity of the β-catenin·Tcf-4 complex. Different mechanisms have been put forward, involving the interaction of PS1 with N- and E-cadherin and β-catenin and the activity of the γ-secretase complex [4], [5], [49]–[51]. We previously studied the effect of a non-processed form of PS1 on β-catenin·Tcf-4 transcription in epithelial cells. In this study, we have extended our analysis to MEFs and compared the effects of processed and non-processed forms of PS1. Ours results indicated that, differently to pPS1, npPS1 has high affinity for β-catenin and for plakoglobin, a protein that is very similar to β-catenin. Moreover, npPS1 potentiates the association of plakoglobin and β-catenin with Tcf-4 and prevents the interaction of the ternary complex PS1-plakoglobin-Tcf-4 with DNA [32]. All these effects do not require the γ-secretase activity, since they are not prevented by L-685,458, a widely used inhibitor of this activity.

When processed, PS1 affects β-catenin·Tcf-4 transcription through a completely different mechanism, in this case dependent on its γ-secretase activity. Unlike npPS1, pPS1 has a detectable association with E or N-cadherin and facilitates the generation of a C-terminal fragment of both proteins consisting of the cytosolic domain (CTF2). It is not yet known whether the interaction of PS1 with cadherins is direct or mediated by other membrane components since the binding experiments have not been performed using pure proteins. Therefore, the lack of interaction of npPS1 with cadherins may be due to its inability to associate with the factor mediating this interaction, maybe because of a hampered localization of npPS1 in the cytoplasmic membrane. This lack of interaction with cadherins might be a more general property of mutant PS1 forms, since many of these mutants show defects in their cell trafficking and, presumably, are not located in the cell membrane [14], [52]. At this respect, it has been recently shown that interaction of pPS1 with N-cadherin and its localization in the plasma membrane is negatively controlled by GSK3β [53]. However, the lack of association of npPS1 with E-cadherin detected in pull-down assays suggests that some intrinsic properties of the non-processed proteins hinder its interaction with the cadherins, either directly or indirectly. In any case, it is possible that the different association of PS1 forms with cadherins has physiological relevance, since binding of pPS1 to N-cadherin facilitates the recruitment of PI3 kinase (PI3K) and the activation of the PI3K/Akt survival pathway [54]. The absence of association of npPS1 with N-cadherin and the lack of activation of this pathway might be responsible for the neurodegeneration observed in cells bearing a mutation that prevents processing

It should be noted that pPS1 affects the stability of E- and N-cadherin, although γ-secretase does not participate in the processing of the full-length protein, but of the CTF1 fragment. Since pPS1 binding competes with that of p120-catenin, it is possible that pPS1 prevents the stabilization caused by this catenin on E-cadherin [55] or, alternatively, potentiates the initial processing of E-cadherin, perhaps facilitating the binding of the responsible protease.

The effect of pPS1 on E-cadherin stability is also demonstrated by the remarkable levels of expression of this protein in MEFs deficient for PS1. Actually these cells present a more epithelioid phenotype than wild type MEFs (data not shown). This unexpected result suggests that in these fibroblasts E-cadherin expression is not exclusively modulated at the transcriptional level, as it is generally considered [56]. At this regard, MEFs present lower expression of the E-cadherin repressor Snail1 (Snail) [56] than cultured fibroblastic cell lines (I.R., A.G.H. and M.D., unpublished observations). Snail1 mRNA is not significantly different in PS1(−/−) MEFs with respect to wild type MEFs, suggesting that down-regulation of this transcriptional repressor does not contribute to the increased levels of E-cadherin expression detected in PS1-deficient MEFs. It is also likely that up-regulated E-cadherin affects the levels of other associated proteins, such as β-catenin and plakoglobin, inhibiting their degradation.

Unlike what happens with E-cadherin, npPS1 associates with β-catenin or plakoglobin much better than pPS1. It has been reported that binding of PS1 to β-catenin would be direct through the 298–380 hydrophilic loop [57], [58]. Since processing of PS1 occurs through the cleavage of a peptidic bond present in this loop, it is likely that it affects the interaction with β-catenin and plakoglobin. This could explain the contradictory results obtained by many authors regarding the interaction of PS1 and β-catenin, an association reported to be either direct or dependent on E-cadherin [4], [51].

Our results totally agree with previous reports indicating that inhibition β-catenin transcriptional activity by pPS1 is dependent on γ-secretase-dependent generation of N-cadherin CTF2, a fragment that induce the destabilization of the transcriptional coactivator CBP [10]. We have extended this conclusion to E-cadherin CTF2, also capable to bind and down-regulate CBP. On the contrary, at least in MEFs (this report) and several epithelial cell lines (not shown), npPS1 does not promote the generation of E- or N-cadherin CTF2. Therefore, npPS1 selectively affects the transcriptional activity of β-catenin·Tcf-4 complex, not affecting other transcriptional factors that also recruit CBP. Consequently, according to our model, mutations in PS1 that prevent processing, such as those shown in Figure 1, C410Y and Δe9, will decrease the activity of the β-catenin·Tcf-4 complex but will not affect, or even up-regulate, other pathways involving CBP, such as those related to CREB, NF-κB or p53 [10], [59], [60]. The effect of npPS1 on β-catenin·Tcf-4 transcriptional activity would be more general than that of pPS1, since the latter will be limited to the transcriptional targets sensitive to CBP. Therefore, this unbalanced transcriptional activity might be contributing to neurodegeneration in FAD. According to this idea, PS1 mutants unable to generate CTF2 but still capable of being processed, may behave less severely than those deficient in processing, since their effect on β-catenin·Tcf-4 transcription would be less robust. Although this model is based on data obtained in cell lines, and should be verified using a more physiological context, as knock-in mouse animal models [61], [62], we consider that it proposes an attractive possibility worthy to be investigated.
