# Correction: Gamma-Secretase-Dependent and -Independent Effects of Presenilin1 on β-Catenin·Tcf-4 Transcriptional Activity

**DOI:** 10.1371/journal.pone.0161515

**Published:** 2016-08-15

**Authors:** Imma Raurell, Montserrat Codina, David Casagolda, Beatriz del Valle, Josep Baulida, Antonio García de Herreros, Mireia Duñach

The authors would like to correct Fig 1C, as errors were introduced in the preparation of this figure for publication. The first 6 lanes in the anti-beta actin 24 hours after transfection panel were duplicated in the 48 hours after transfection panel. The authors have repeated the analysis and provide the revised Fig 1 with a new panel C and the underlying data here.

**Fig 1:**
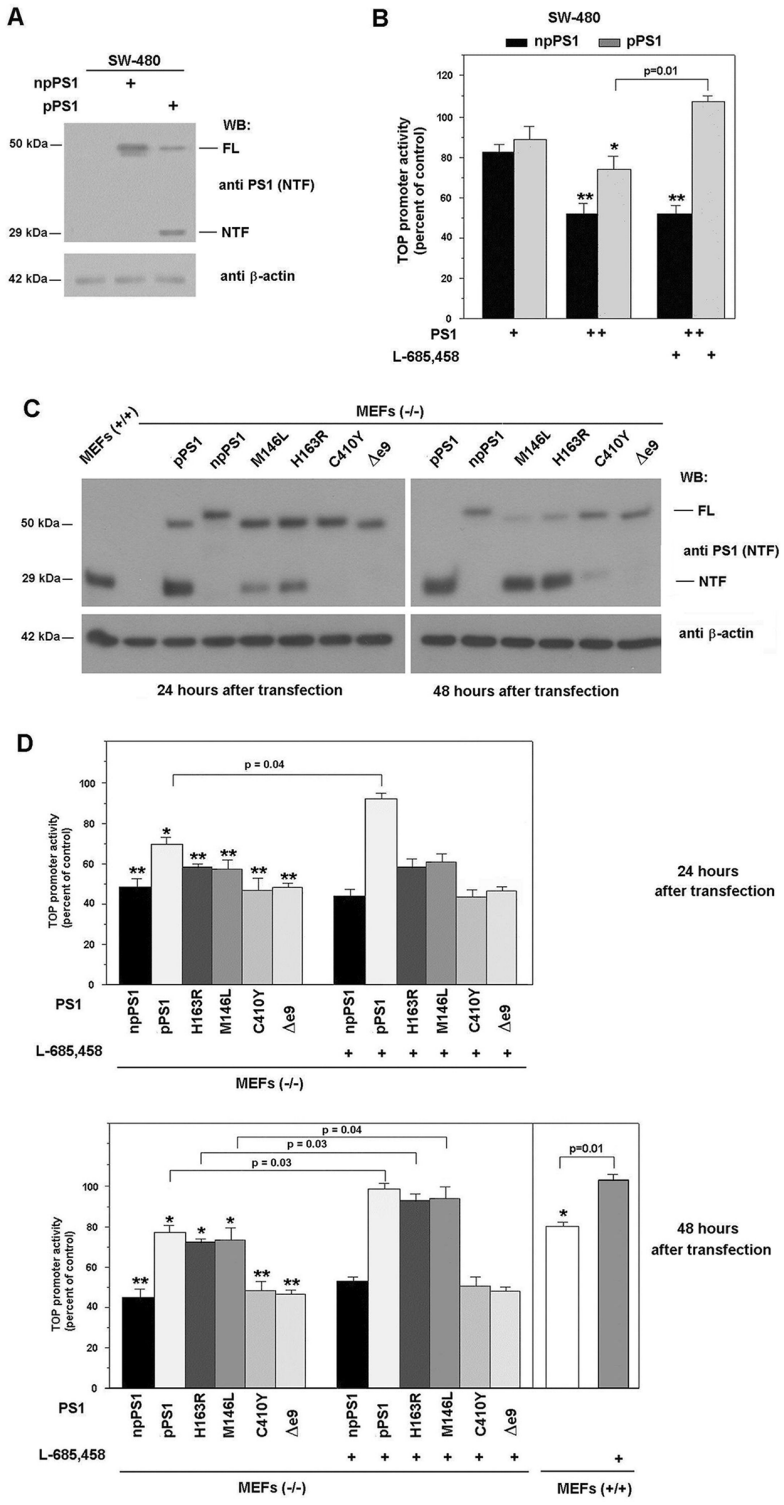
Processed and non-processed PS1 block β-catenin·Tcf-4-dependent transcription differently. SW-480 (A,) and MEF PS (−/−) cells (C) were transfected with 5 μg of pcDNA3 plasmid containing either *Myc/His*-tagged PS1 (npPS1), wild-type PS1 (pPS1), the indicated mutants, or empty vector as a control. After 48 hours (A) or at the indicated times (C), cell extracts were prepared as described in *Materials and Methods*. 50 μg of untransfected or transfected total cell extracts were analyzed by SDS-PAGE and Western blot with antibodies anti-PS1 (amino acids 1–65) and anti-β-actin as a control (A and C). In panels B and D cells SW-480 and MEFs were cotransfected with 150 (+, in B) or 300 ng (++, in B; and D) of pcDNA-3 containing npPS1, pPS1, or, the indicated mutants, plus TOP-FLASH (50 ng) and pTK-*Renilla* (10 ng) luciferase plasmids. Relative luciferase activity was determined with a dual luciferase reporter assay system 48 hours after transfection (B) or at the indicated times (D), and the result was normalized using the *Renilla* luciferase activity for each sample. Percentage activity was calculated by comparing levels of luciferase activity with levels after transfection of the empty plasmid alone. 5 μM γ-secretase inhibitor L-685,458 (Calbiochem) was added to the medium for the last 24 h (panels B and D). Values are the average +/− S.D. of three-four experiments performed in triplicate. One asterisk (*) indicates p<0.05; two asterisks (**) p<0.01; in the rest of the comparisons, the value of p is presented.

The authors would also like to provide additional clarifications for Fig 3. In the published Fig 3, the two bands from panels A and B corresponding to plakoglobin are the same and correspond to the plakoglobin analysis in MEFs PS1 WT in both lanes. The authors decided to present the data from the Western blot split in two panels; in panel A, the differences between PS1 WT and KO and in panel B, the effect of the inhibitor on PS1 WT. The experiments were repeated four times. Panel A was assembled from experiments comparing MEFs KO, control and control plus 1 μM L-685,485; panel B combining those initial experiments (plakoglobin, β-catenin) and experiments involving the control plus two concentrations of L-685,485, 1 and 5 μM.

The authors have replicated the analysis and provide a revised Fig 3 with its underlying images here. The updated Fig 3B includes the three conditions (control and two concentrations of L-685) for all proteins (the 5 μM condition was lacking for plakoglobin and β-catenin in the originally published Fig 3).

**Fig 3:**
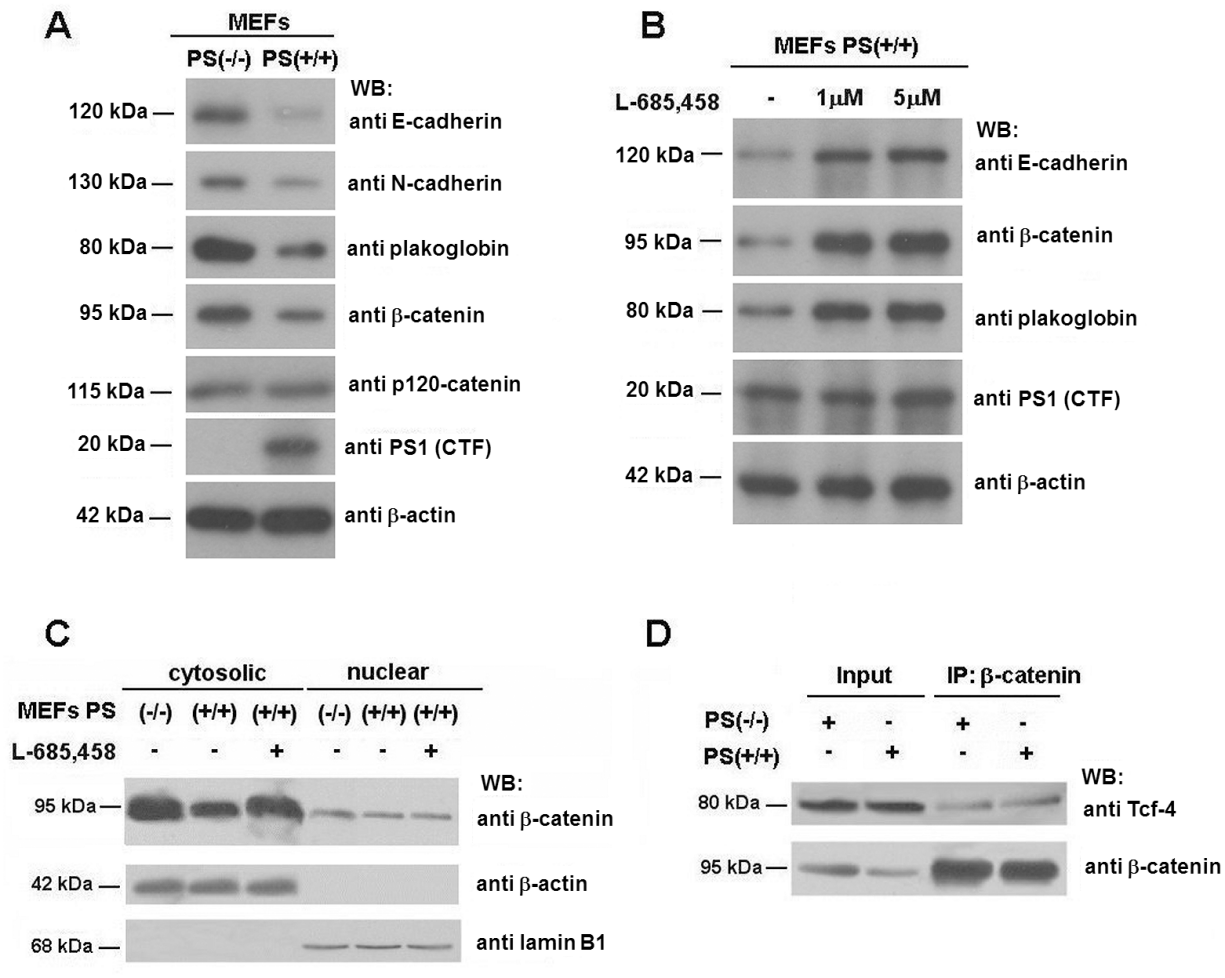
PS depletion increases protein levels of adherens junction-associated proteins. (A) 50 μg of MEF PS (−/−) and PS (+/+) total-cell extracts were analyzed by Western blotting with the indicated antibodies. (B) MEF PS (+/+) cells were incubated with 1–5 μM γ-secretase inhibitor L-685,458 for 24 h. Then, total cell extracts were prepared as described in *Experimental Procedures* and analyzed by SDS-PAGE and Western blot with the indicate antibodies. No effect of the γ-secretase inhibitor was detected in PS/−/−) cells on the levels of the studied proteins (not shown). (C) Cytosolic and nuclear fractions of MEF PS (−/−) and PS (+/+) cells were prepared as described. When indicated, MEF PS (+/+) cells were incubated with 5 μM γ-secretase inhibitor L-685,458 for 24 h. 50 μg of each fraction was analyzed by Western blotting with anti-β-catenin, anti-β-actin and anti-laminB1. (D) 800 μg of MEF PS (−/−) and PS (+/+) total-cell extracts were immunoprecipitated with an anti-β-catenin MAb, and analyzed by Western blot with specific antibodies against Tcf-4 and β-catenin. In the Input lane, a sample corresponding to 7% of the total cell extracts used for the assay was loaded.

The authors confirm that these changes do not alter the findings reported in the article.

## Supporting Information

S1 File Underlying images for updated Figs 1C and 3A and 3B. (ZIP)
