# Supplementary figures and images for: Correction: Gamma-Secretase-Dependent and -Independent Effects of Presenilin1 on β-Catenin·Tcf-4 Transcriptional Activity
Source: PLoS One. 2016 Aug 15;11(8):e0161515. doi: 10.1371/journal.pone.0161515 (PMC4985063; doi:10.1371/journal.pone.0161515)

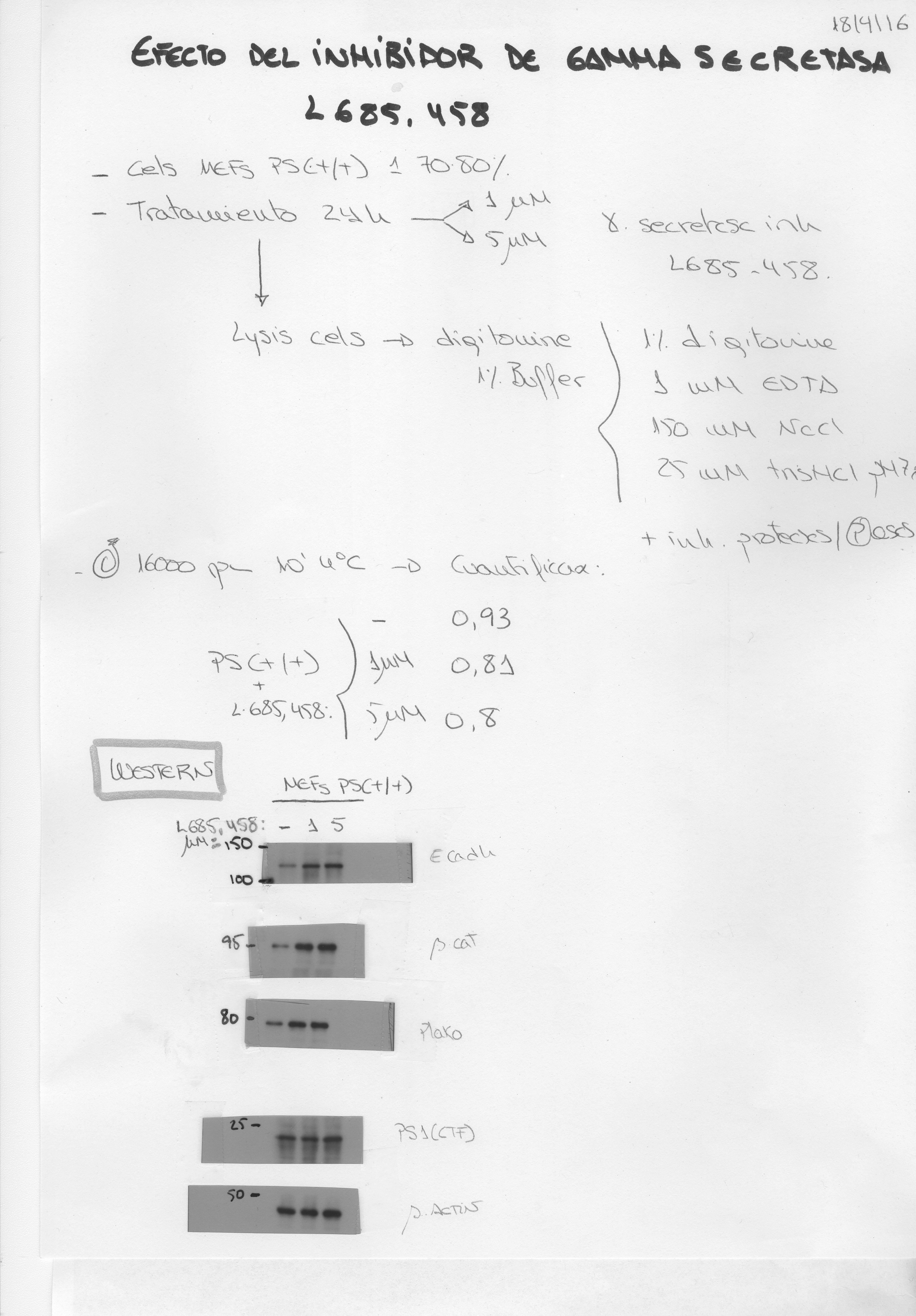

Supplement: S1 File — (ZIP) [file pone.0161515.s001.zip › Fig 3 panel 3B MEFs PS new exp.jpg]

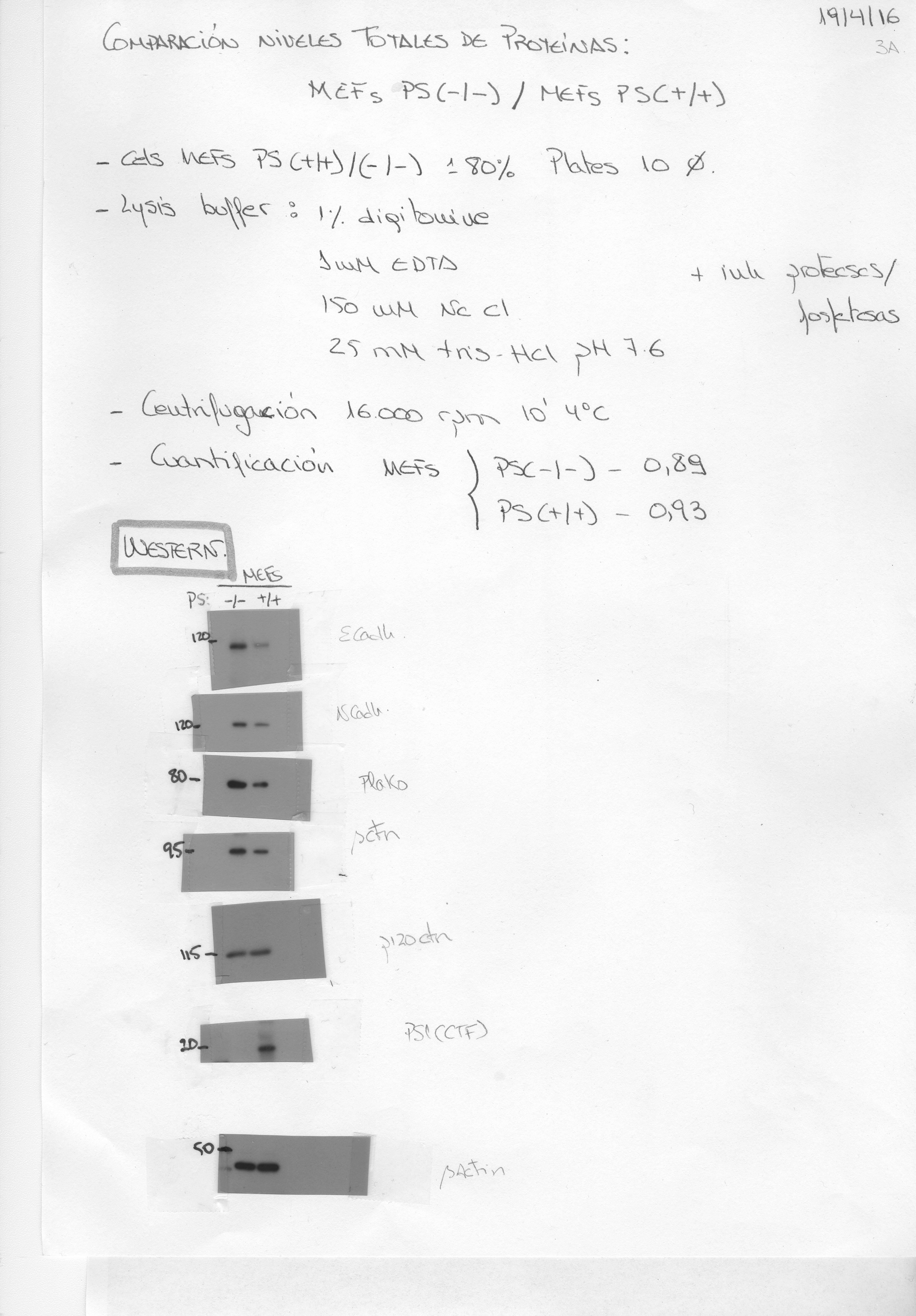

Supplement: S1 File — (ZIP) [file pone.0161515.s001.zip › Fig 3 panel 3A MEFs PS new exp.jpg]

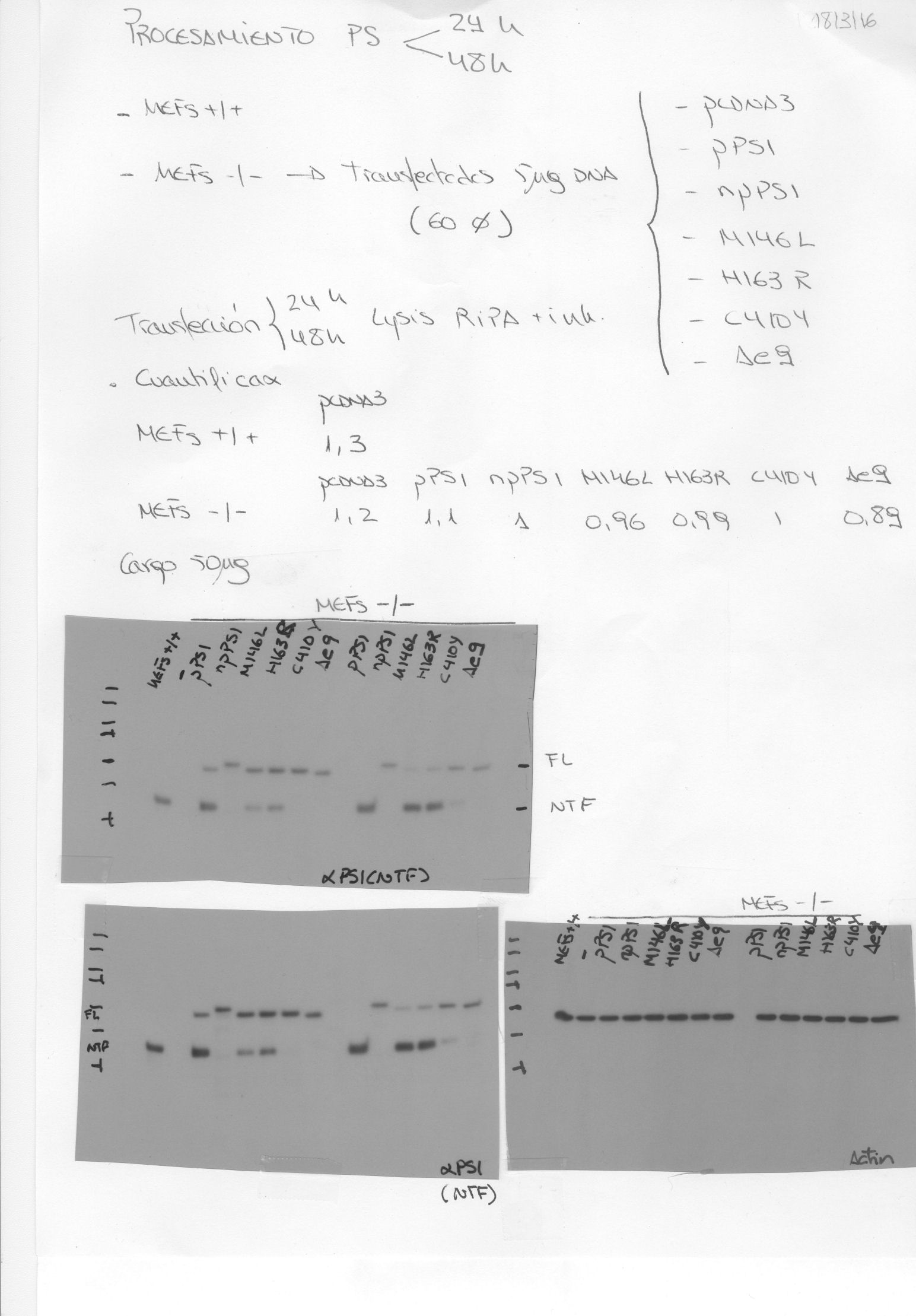

Supplement: S1 File — (ZIP) [file pone.0161515.s001.zip › Data for Fig 1.jpg]
